# IL-20 in Acute Kidney Injury: Role in Pathogenesis and Potential as a Therapeutic Target

**DOI:** 10.3390/ijms21031009

**Published:** 2020-02-03

**Authors:** Tian-Yu Lin, Yu-Hsiang Hsu

**Affiliations:** 1 Institute of Clinical Medicine, College of Medicine, National Cheng Kung University, Tainan 70101, Taiwan; lintanguin@hotmail.com; 2 Research Center of Clinical Medicine, National Cheng Kung University Hospital, College of Medicine, National Cheng Kung University, Tainan 70101, Taiwan

**Keywords:** IL-20, acute kidney injury, chronic kidney disease

## Abstract

Acute kidney injury (AKI) causes over 1 million deaths worldwide every year. AKI is now recognized as a major risk factor in the development and progression of chronic kidney disease (CKD). Diabetes is the main cause of CKD as well. Renal fibrosis and inflammation are hallmarks in kidney diseases. Various cytokines contribute to the progression of renal diseases; thus, many drugs that specifically block cytokine function are designed for disease amelioration. Numerous studies showed IL-20 functions as a pro-inflammatory mediator to regulate cytokine expression in several inflammation-mediated diseases. In this review, we will outline the effects of pro-inflammatory cytokines in the pathogenesis of AKI and CKD. We also discuss the role of IL-20 in kidney diseases and provide a potential therapeutic approach of IL-20 blockade for treating renal diseases.

## 1. Introduction

The kidney functions as a filter to remove metabolic waste products and toxic substances as well as excess water from the body and thus maintains the balance of the body fluids, electrolytes, and blood pressure. The nephron is the functional unit of the kidney and is composed of the glomerulus and renal tubular. Renal injury results in nephron loss and then causes tubular atrophy and interstitial fibrosis. Renal dysfunction causes nitrogenous wastes accumulation in the body, resulting in poisoning (e.g., uremia). Kidney failure can be divided into acute kidney injury (AKI) and chronic kidney disease (CKD) based on its duration. The former may be cured as long as it is properly treated, while the latter is usually irreversible. Tissue damage can be repaired and regenerated to restore the normal structure and function. However, if the injury is very severe or prolonged, repair may be incomplete (maladaptive repair), which leads to tissue dysfunction and fibrosis. Persistent inflammation, increased numbers of myofibroblasts, and extracellular matrix (ECM) accumulation are usually observed in many kinds of kidney diseases [1].

ECM are produced from several cells, such as interstitial fibroblasts, mesangial cells, epithelial cells, and endothelial cells and composes by fibrous proteins and glycosaminoglycans [2]. Moreover, these cells contribute to renal inflammation by producing different kinds of cytokines and chemokines. In diabetes-induced CKD, also called diabetic nephropathy (DN), both metabolic stimuli and reactive oxygen species (ROS) production regulate gene expression and transcription factor activation, leading to impaired renal function and structure. The alterations of renal structure include thickening of the glomerular basement membrane (GBM), capillary, and tubular basement membrane and reduction of glomerular endothelium fenestration, expansion of the mesangium and loss of podocytes [3,4,5].

IL-20 is a member of IL-10 family, which includes IL-10, IL-19, IL-22, IL-24, IL-26, IL-28, and IL-29. Many studies have demonstrated that IL-20 is associated with several inflammatory diseases, such as rheumatoid arthritis, atherosclerosis, cancer, and liver fibrosis via regulating cytokines and chemokines. In vivo experiments also showed that blocking IL-20 with specific antibodies can reduce inflammation and improve disease progression. Previous studies indicated that IL-20 and its receptors are increased in the kidneys of mice with acute renal failure (ARF), which might cause apoptosis and necrosis in tubular epithelial cells by activating caspase-9. Besides, the serum level of IL-20 is elevated in patients with CKD and DN. IL-20 upregulates TGF-β1 expression in proximal tubule epithelial cells and promotes IL-1β expression under hypoxic conditions. IL-20 also regulates TGF-β1 production in interstitial fibroblasts. The expression of MCP-1, TGF-β1, MMP-9, and VEGF is upregulated in IL-20-treated podocytes [6]. In this review, we will discuss the role of cytokines in the progression of kidney diseases AKI, CKD, and DN. In the end, we will summarize the impact of IL-20 in the renal lesion and discuss the effect of IL-20 blockade in kidney disease therapy.

## 2. Acute Kidney Injury (AKI)

Acute kidney injury (AKI), previously known as ARF, is characterized by a rapid loss of kidney function within a few hours or days. AKI is common in hospitalized patients with kidney transplantation, trauma, and sepsis and is associated with poor prognosis, high morbidity, and mortality. The mortality rate of AKI in intensive care unit (ICU) patients ranges from 20% to 50% [7,8]. The etiologies of AKI can be classified as pre-renal, intrinsic, and post-renal. Pre-renal AKI is the most common cause and occurs when blood flowing to the kidney is reduced. Intrinsic AKI refers to damage to renal structures, including vascular, glomerular, interstitial, and tubular. Post-renal AKI is usually caused by urinary tract obstruction. Pre-renal and post-renal AKI may eventually develop into intrinsic renal disease. Diagnostic criteria for AKI are based on serum creatinine level (SCr) and urine output. Kidney Disease: Improving Global Outcomes (KDIGO) defined AKI as the following: increase in SCr by ≥ 0.3 mg/dL (≥ 26.5 µmoL/L) within 48 h or increase in SCr to ≥ 1.5 times baseline which has occurred within the prior 7 days; or urine volume < 0.5 mL/kg/h for 6 h [9].

AKI usually occurs if insufficient blood is flowing through the kidney (ischemia) or toxins are overloaded in the kidney (nephrotoxins). The injury site of AKI is mainly in the proximal tubule. Tubular necrosis and inflammatory cell infiltration are observed in the early progression of AKI [10,11]. Necrotic tubular endothelial cells initiate early inflammatory responses and promote the infiltration of immune cells. In the early stage of AKI, neutrophils first rapidly accumulate and adhere to endothelium by adhesion molecules such as P-selectin and intracellular adhesion molecule-1 (ICAM-1) and release cytokines [12]. ICAM-1, IL-1, and TNF-α mRNA levels are increased within 1 h after ischemia/reperfusion. Monocytes and macrophages, following neutrophils, migrate into injury sites and remove debris of dying renal cells and neutrophils. Forbes et al. observed that monocyte/macrophage significantly increases at 4 days after ischemic injury [13]. Cellular debris is also cleared by dendritic cells and dedifferentiated epithelial cells. Surviving epithelial cells dedifferentiate and proliferate to replace the injury cells for restoring the integrity of the tubular epithelial cell layer. Several pieces of evidence display that inhibition of leukocyte (neutrophil, monocyte, and macrophage) reduces tubular necrosis and inflammation [14,15,16].

## 3. Chronic Kidney Disease (CKD)

CKD is defined as kidney damage or glomerular filtration rate (GFR) < 60 mL/min/1.73 m^2^ lasting for more than 3 months with implications for health. It is estimated that more than 700 million people have CKD [17]. The effect of AKI and CKD is bidirectional [18]. Individuals who survive after AKI have a high risk of developing CKD and hastened development of end-stage renal disease (ESRD) because of incomplete repair (maladaptive repair), which leads to vascular rarefaction, tissue fibrosis, and chronic inflammation [19,20,21,22]. Maladaptive repair is characterized by interstitial fibrosis and persistent inflammation. Severe or recurrent injury leads to cell-cycle arrest in tubular epithelial cells, which fail to restore damaged renal structures. Yang et al. investigated the cell-cycle of tubular epithelial cells arrested in the G2/M phase and upregulated fibrogenic factors TGF-β1, collagen-1, and collagen-4 via c-Jun N-terminal kinase (JNK) signaling in AKI models [1]. The macrophage phenotype is important for repair progression in kidney diseases. Macrophage phenotype is traditionally divided into M1 (pro-inflammatory) and M2 (anti-inflammatory). In the early stage of the repair process, M1 macrophages are dominant to clear cellular debris. Macrophages switch from M1 to M2 to help tissue regeneration by enhancing tubular cell proliferation in the late stage. However, arrested cells cause macrophage to stay as the M1 phenotype through releasing pro-inflammatory cytokines. Besides, glomerular mesangial cells also acquire a myofibroblast phenotype and play a role in renal fibrosis. CKD is one of the risk factors for AKI development. Patients with a baseline GFR of 45–59, 30–44, and < 30 mL/min had a relative risk for AKI of 2.9, 6.2, and 18.3, respectively [23,24,25].

Some chronic diseases that cause renal dysfunction include systemic lupus erythematosus (SLE), diabetes mellitus, and hypertension, which lead to lupus nephritis, diabetic nephropathy, and hypertensive nephropathy, respectively. Additionally, some analgesic medications such as aspirin, phenacetin, and nonsteroidal anti-inflammatory drugs lead to renal papillary necrosis and chronic interstitial nephritis [26]. Lupus nephritis is a frequent complication of SLE, an autoimmune disease, and is characterized by autoantibodies and complement deposition in the kidneys, which lead to inflammation and loss of kidney function. The morbidity and mortality rates of lupus nephritis account for approximately 60% of SLE patients [27,28,29]. Hypertensive nephropathy is a kidney disorder that is associated with hypertension. Hypertension results in arteriolosclerosis and damage of blood vessel lining, including thickening of blood vessel walls and narrowing of preglomerular arteries and arterioles openings, thereby reducing glomerular blood flow. Insufficient blood flow can cause glomerular ischemia and hyperfiltration, which can lead to tubular atrophy, interstitial fibrosis, and changes in glomerular structure [30]. IgA nephropathy (IgAN), also known as Berger’s disease, is the most common glomerulonephritis worldwide and is caused by the accumulation of the IgA in the glomerulus [31]. IgA predominantly deposits in the mesangium of the kidney, which triggers mesangial cell proliferation and production of pro-inflammatory cytokines and pro-fibrotic mediators to mediate tubulointerstitial injury and fibrosis. Up to 30% of patients with IgAN will develop ESRD within 20 years [32,33].

## 4. Diabetic Nephropathy

Diabetic nephropathy (DN), also known as diabetic kidney disease (DKD), is a complication of diabetes mellitus and is the most frequent cause of ESRD. Diabetes leads to alterations in metabolism and hemodynamics and reactive oxygen species (ROS) generation. Hyperglycemia activates protein kinase C (PKC) pathway and increases advanced glycated proteins (AEG) generation, mitochondrial oxidative phosphorylation, and ROS production [34]. PKC activation is associated with impairment of renal function and structure, including glomerular filtration, ECM accumulation, and cell apoptosis [35]. ROS disrupts the cellular structure by destroying lipid, protein, and DNA. ROS also causes podocyte apoptosis or detachment [36]. Podocytes are highly specialized epithelial cells with complex cellular structures called foot processes and constitute the epithelial layer of Bowman’s capsule and wrap around capillaries. The foot processes of podocytes form filtration slits to regulate GFR. Thus, podocyte loss resulting in albuminuria increases [37,38]. Hyperglycemia also increases oxygen consumption to cause renal hypoxia. Several studies show that high glucose increases inflammatory cytokine production, including that of TNF-α, MCP-1, IL-1, IL-6, and IL-8 in renal tubular cells, podocytes, and mesangial cells, which synergistically aggravate local and systemic inflammation in kidney.

## 5. Inflammation and Kidney Disease

Inflammation response is mediated by several types of immune cells and essential for pathogen elimination, tissue repair, and regeneration. However, the excessive inflammatory response may result in tissue damage, fibrosis, and functional loss in many diseases such as cancer and kidney diseases. Immune cells release soluble mediators, cytokines, and chemokines, which impaire biological function and cellular structure of renal cells. In addition to immune cells, renal cells such as podocytes, mesangial cells, and tubular cells also release many cytokines to promote kidney injury. It has been determined that pro-inflammatory cytokines and chemokines such as TNF-α, IL-1β, IL-6, IL-8, and MCP-1 are involved in the development of kidney diseases.

## 6. MCP-1(CCL2)/CCR2

Serum and urinary MCP-1 levels are significantly elevated in patients with kidney diseases [39,40]. MCP-1 and its receptor CCR2 are important for recruiting monocyte/macrophage into the kidney in patients with AKI and CKD. Several cytokines induce renal tubular epithelial cells and mesangial cells to secrete MCP-1, which in turn stimulates ICAM-1 expression to promote leukocyte retention [41,42,43,44,45,46]. High glucose and AEG stimulation also induce MCP-1 expression to enhance fibrosis-associated factors production through activation of nuclear factor-κB (NF-κB) [47,48,49,50,51,52]. Podocytes’ exposure to high glucose activates TGF-β-dependent MCP-1 expression, which in turn induces apoptosis and increases permeability [53,54]. Blockade of MCP-1/CCR2 signaling ameliorates glomeruli hypertrophy, interstitial fibrosis, and tubular atrophy [55,56,57,58,59]. MCP-1-deficient mice with renovascular hypertension have reduced renal damage and inflammation [55]. In the DN animal model, the numbers of macrophages in the kidney are decreased in MCP-1-deficient mice. MCP-1 inhibition decreases the production of TNF-α, IL-6, and TGF-β and ameliorates podocyte function and albuminuria. CCR2 antagonist also showed an improvement of mesangial expansion and GBM thickening [56]. However, a previous study indicated that MCP-1 plays a protective role in the early phase of ischemia/reperfusion injury (IRI)-induced AKI progression. Stroo et al. determined that MCP-1 deficiency reduces survival and increases renal damage after IRI [60]. Furthermore, in MCP-1 knockout mice with IRI-AKI, macrophage type moves towards more M1 (pro-inflammatory) phenotype. It is found that MCP-1 activates M2-type macrophage polarization, which promotes tubule epithelial cell proliferation in the repair phase of AKI. Therefore, there may be a need to consider whether the target MCP-1 is suitable for the treatment of IRI-AKI.

## 7. IL-8/CXCL8

IL-8 acts as a chemoattractant, which predominantly drives neutrophils to inflammation sites through CXCR1 and CXCR2. Pro-inflammatory cytokines IL-1 and TNF-α stimulate IL-8 expression in human mesangial cells and proximal tubule cells [45,61,62,63]. IL-8 stimulates ICAM-1 production in human proximal tubule cells through the p38 pathway [64,65]. IL-8 is increased in the podocytes and endothelial cells of kidneys in patients with glomerulonephritis. Administration of IL-8 antibody ameliorates renal function in rabbits with glomerulonephritis [66]. G31P, a CXCR1/CXCR2 inhibitor, ameliorates sepsis-induced renal damage through reducing renal cell apoptosis and improves inflammation via reducing IL-1β, IL-6, and TNF-α expression, and neutrophil infiltration. Similar results are also confirmed in cisplatin-induced AKI model [67,68]. G31P also showed the reduction of renal structure change, including GBM thickening, mesangial expansion, collagen deposition, and podocyte loss in diabetic mice [69]. In addition, G31P downregulates TGF-β, CTGF, and fibronectin expression as well as upregulates MMP-2 and MMP-9. G31P inhibits renal inflammation by reducing IL-1β, IL-6, and TNF-α expression and decreasing macrophage infiltration. G31P also inhibited high-glucose-induced TNF-α and TGF-β expression in mesangial cells by reducing the phosphorylation of JAK2, STAT3, and ERK1/2 [69].

## 8. TNF-α

The role of TNF-α in kidney disease was first discovered in 1989 by Bertani et al. [70] TNF-α causes renal damage through inducing apoptosis in epithelial cells, tubular cells, and mesangial cells, which can be inhibited by blockade of TNF-α [71,72,73]. TNF-α stimulates MCP-1 and IL-8 expression as well as adhesion molecular ICAM-1 expression in mesangial cells, tubular epithelial cells, and podocytes to promote neutrophil and monocyte infiltration. TNF-α also increases ROS production in mesangial cells. In addition, TNF-α is correlated with urinary albumin excretion [74,75,76]. TNF-α induces the loss of glomerular endothelial cell fenestration and then causes GFR decrement and albumin leakage. Inhibition of TNF-α decreases macrophage recruitment and inhibits G-MCSF, keratinocyte-derived cytokine (KC), and MCP-1 levels and reduces albuminuria in DN animal model [77]. Several studies demonstrated that systemic TNF-α inhibition attenuates renal function and inflammation in many kidney disease animal models [78,79,80]. However, Wen et al. found that TNF-α deletion in T lymphocytes increases IL-17A expression and the numbers of CD4^+^ and CD8^+^ T cells in the kidney with nephrotoxic nephritis. T cell-derived TNF-α protects against renal injury and fibrosis in mice with nephrotoxic nephritis [81].

## 9. IL-1β

IL-1β is mainly secreted from infiltrating leukocytes (dendritic cells, macrophages, and neutrophils) [82]. Podocytes are the only renal intrinsic cells in the kidney from CKD patients that secrete IL-1β, but numerous in vitro experiments indicate that other renal cells can release IL-1β under some stimulus [83,84,85,86]. Macrophage-derived IL-1β promotes cell proliferation and induces fibronectin production in fibroblasts and mesangial cells [87,88,89]. IL-1β increases the expression of IL-6 and IL-8 in primary human renal fibroblasts and mesangial cells [89,90,91]. IL-1β induces ROS production and fibrotic factor expression, such as that of TGF-β, collagen I, and fibronectin in tubular epithelial cells [85,92,93]. Additionally, IL-1β promotes tubular epithelial cells to transdifferentiate into fibroblast-like phenotype, which is critical in the progression of renal fibrosis [94,95]. Faubel et al. observed that the level of IL-1β is increased in cisplatin-induced AKI mice. Shahzad et al. identified that IL-1β gradually increases in DN progression of db/db mice, which implies that IL-1β is involved in the development of DN [96]. IL-1β deficiency ameliorated renal function and inflammation in mice with nephrotoxic nephritis [97,98]. IL-1β activation is mediated by caspase-1. NLRP3 inflammasome, a multiple protein complex composed of the sensor molecule NLRP3, the adaptor protein ASC, and pro-caspase-1, regulates caspase-1 activation. Caspase-1 is associated with pyroptosis, a programmed lytic cell death that is different from apoptosis and necrosis, which accelerates tubular epithelial cell death and is the most common cause of AKI [99,100,101,102]. NLRP3 inflammasome can be activated by glucose in glomerular endothelial cells and podocytes [96]. A few in vivo studies have shown that NLRP3 deficiency reduces renal inflammation, ECM accumulation, and fibrosis [86,92,103,104,105]. In addition to IL-1β, NLRP3 inflammasome can also trigger IL-18 maturation, which is associated with the pathogenesis of various kidney diseases [105,106,107,108].

## 10. IL-6

IL-6 signals through two distinct pathways to exert its cellular effects. (1) IL-6 acts on membrane-bound IL-6R (mbIL-6R) and then triggers gp130 to activate subsequent downstream signaling (classical signaling). (2) IL-6 forms dimer with soluble IL-6R (sIL-6R) and then binds to gp130 to initiate signal transduction (trans-signaling). IL-6 can be produced by renal resident cells, including tubular epithelial cells, podocytes, and mesangial cells under some stimuli such as TNF-α and IL-1β [109,110,111,112]. However, the expression of IL-6R in renal cells is limited. Except for podocytes, other renal resident cells do not express mbIL-6R, which implies that IL-6 stimulates these cells via trans-signaling [113]. IL-6 induces collagen I expression in mouse proximal tubular epithelial cells via STAT3 phosphorylation. IL-6 plays a role in mesangial cell proliferation, which is associated with glomeruli hypertrophy and stimulates MCP-1 expression [112,114,115]. In addition, IL-6 is involved in high-glucose-induced podocyte apoptosis through regulating caspase-3 and caspase-9 activation. Furthermore, IL-6 increases p21 and p27 production, which results in cell cycle arrest in podocytes [116]. IL-6 enhances the fibrotic response via TGF-β, collagen I, and collagen IV in IRI-AKI model. IL-6 deficiency ameliorates renal function and decreases neutrophil infiltration in IRI- and HgCl_2_-induced AKI models [117,118]. IL-6 blockade improves renal inflammation in IRI mice by reducing TNF-α and IL-1β production and decreasing ICAM-1 and P-selectin expression, which contribute to neutrophil infiltration. Tocilizumab (TCZ), a humanized IL-6R antibody, attenuates albuminuria and glomerular hypertrophy as well as suppresses NLRP3 inflammasome activation in diabetic mice and mice with lupus nephritis. Inhibition of IL-6 trans-signaling downstream transcription factor protects against renal fibrosis and attenuates inflammation [119]. These findings reflect the fact that IL-6 contributes to renal inflammation and declining renal function as well as disrupts glomerular structure. However, some studies indicate that IL-6 has anti-inflammatory effects in kidney diseases [117,120]. IL-6 administration protects against nephrotoxic nephritis. Enhanced IL-6/sIL-6 axis protects against HgCl_2_-induced AKI via reducing oxidative stress. Therefore, IL-6 might have both pro-inflammatory and anti-inflammatory functions in different kidney diseases.

## 11. Transforming Growth Factor-β (TGF-β)

TGF-β exerts biological functions, including cell proliferation, differentiation, and apoptosis in different types of cells. There are three isoforms of TGF-β (TGF-β1, TGF-β2, and TGF-β3). Numerous studies demonstrate that TGF-β1 is involved in renal fibrosis and causes the excessive accumulation of ECM components in renal cells through downregulating MMPs and upregulating TIMPs. TGF-β1 induces fibroblast proliferation via basic fibroblast growth factor (bFGF) and increases osteopontin and collagen I expression in fibroblasts in unilateral ureter obstruction (UUO) mice [121]. Moreover, TGF-β1 is involved in tubular epithelial-to-myofibroblast transition (EMT), which contributes to more ECM accumulation and regulates macrophage infiltration to mediate tubulointerstitial fibrosis [122,123]. Furthermore, elevated glucose levels stimulate TGF-β1 production through the PKC pathway to upregulate Glut4 expression, which results in increasing cellular glucose uptake and accelerating the progression of diabetic kidney. In addition to promoting fibrosis, TGF-β also induces caspase-3-dependent podocyte apoptosis by activating the mTOR pathway [124,125]. Based on the role of TGF-β1 in fibrogenesis, several strategies for inhibiting TGF-β1 have proven to alleviate renal fibrosis effectively. Ziyadeh et al. demonstrated that treatment with TGF-β1 antibody prevents renal fibrosis in DN mice [126]. Gewin et al. showed that HgCl_2_-induced apoptosis of proximal tubule epithelial cells is mitigated in TGF-β1 receptor deficiency mice [127]. Several clinical trials using TGF-β antibodies for treating focal segmental glomerulosclerosis and DN have been explored. However, completely blocking TGF-β signaling may cause severe side effects according to the anti-inflammatory effect and anti-tumor role of TGF-β1 [128]. Hence, other groups target the downstream of the TGF-β1 signaling pathway. TGF-β1 binds to receptor complexes, TGF-β type I receptor (TβRI), and TGF-β receptor type II (TβRII) and then phosphorylates Smad2 and Smad3 to regulate fibrogenic genes. Smad3, but not Smad2, is recognized as an important factor in the EMT process. It is confirmed that Smad3 deficiency inhibits fibrosis and improves renal function in different CKD models [129,130]. Smad7, an inhibitory Smad, negatively regulates TGF-β1 signaling by TGF-β receptor competition and degradation; therefore, Smad7 overexpression protects against TGF-β1-induced fibrosis in kidney [131,132,133,134].

## 12. Hypoxia in Kidney Disease

Several research groups postulated that hypoxia is critical in the advancement of AKI to CKD and ESRD [135,136,137]. Healthy kidneys receive over 20% of the cardiac output and comprise roughly 10% of total oxygen consumption. In healthy kidneys, renal blood flow and oxygen level in the medulla are lower compared to those in the cortex [138]. Thus, the medulla is susceptible to oxygen change. Hyperglycemia increases oxygen consumption in DN [139]. Basile et al. demonstrated that capillary density around the tubules is reduced by about 30–50% in ischemic AKI rats. Reduction of renal vascular density activates the hypoxia-dependent pathway to exacerbate inflammation and tissue fibrosis [140,141]. Mazzali and colleagues place rats in a hypobaric chamber for 24 days to mimic chronic hypoxia. They found that chronic hypoxia increases blood pressure and serum uric acid due to changes in the kidney, including arteriolopathy, glomerular hypertrophy, mild inflammation, and fibrosis [142]. A previous study indicated that hypoxia promotes fibrotic progression by inducing collagen and TGF-β1expression as well as myofibroblast differentiation [143].

Hypoxia-inducible factor-1 (HIF-1), composed of HIF-1α with HIF-1β, is thought to play a role in kidney disease. HIF-1α is activated in response to hypoxia stimulation and is unstable under normal oxygen concentration. Prolyl hydroxylase domain (PHD) proteins hydroxylate HIF-1α, which triggers ubiquitination and proteasomal degradation. Pretreatment of cobalt chloride, PHD inhibitors, reduces model [144]. Furthermore, cobalt markedly reduces renal AEG contents and TGF-*β* in the DN model [145]. Since cobalt chloride is harmful in the human body, several PHD inhibitors are synthesized and used in the clinical trials [146,147,148,149].

HIF-1α activation is time-dependent. HIF-1α is rapidly induced at the beginning of hypoxia and then disappears within 72 h. This is because HIF-1α mRNA is unstable under prolonged hypoxic conditions [150,151]. Rosenberger et al. identified that HIF-1α expression is undetectable at 48 h after AKI [152]. In addition, chronic hypoxia may activate PHD, which in turn promotes HIF-1α degradation [153,154].

## 13. IL-20

Blumberg et al. identified sequences of IL-20 by EST databases in 2001. They found that overexpression of IL-20 leads to abnormal differentiation in epidermal keratinocytes and causes the death of newborn mice. IL-20 activates JAK/STAT signal pathway through two heterodimeric receptors IL-20R1/IL20R2 and IL-222R1/IL-20R2. IL-20 is mainly secreted by immune cells such as monocytes, macrophages, dendritic cells, and lymphocytes. Under the pathological condition, IL-20 is also expressed in various types of cells, including endothelial cells, synovial fibroblasts, chondrocytes, and osteoclasts. Previous studies reported that IL-20 is involved in several inflammatory diseases like psoriasis, rheumatoid arthritis (RA), atherosclerosis, osteoarthritis (OA), and stroke (Table 1). IL-20, as a pro-inflammatory mediator, regulates cytokine and chemokine expression in different types of cells. In RA, IL-20 activates the ERK-1/2 pathway to stimulate MCP-1, IL-6, and IL-8 in synovial fibroblasts as well as promotes neutrophil migration [155]. In OA, IL-20 induces TNF-α and IL-1β expression in synovial fibroblasts and increases IL-6 and MCP-1 in chondrocytes [156]. IL-20 promotes TNF-α, IL-1β, and MCP-1 expression and increases ROS production in oral cancer cells (OC-3 and OEC-M1) [157].

In addition to the effects on pro-inflammatory responses, IL-20 is also involved in angiogenesis and fibrosis. IL-20 promotes angiogenesis by upregulating angiogenesis factors bFGF, VEGF, and MMP-2 to enhance proliferation, migration, and vascular tube formation of endothelial cells (human umbilical vein endothelial cells (HUVECs) and human dermal microvascular endothelial cells (HMECs)) [158,159]. IL-20 is expressed in liver tissues of patients with liver cirrhosis. IL-20 is increased in mice with CCl4-induced liver fibrosis. IL-20 induces TGF-β1 expression and arrests the cell cycle in the G0/G1-phase by upregulating p21 production in hepatocyte Clone-9 cells. IL-20 upregulates TNF-α, TGF-β1, and Col-I mRNA transcripts in hepatic stellate cells (HSCs) [158]. Moreover, IL-20 acts on lung epithelial cells (MLE-12) and promotes fibronectin-1 and α-SMA protein levels [160]. Hypoxia, a critical factor in the pathogenesis of kidney disease, also stimulates IL-20 expression in different type cells (HaCaT cells, HEK293 cells, chondrocytes, glioblastoma cells, and HUVECs).

## 14. IL-20 in AKI

The leading causes of AKI are ischemia and nephrotoxicity. We previously showed that IL-20 and its receptors were upregulated in the kidneys of IRI- and HgCl_2_-induced AKI models, which implies that IL-20 may play a role in AKI. IL-20 not only upregulates TGF-β1 expression but also promotes cell death by activating caspase-9 in human proximal tubular epithelial cells (HK-2). Under hypoxic conditions, IL-1β transcript is increased by IL-20 in HK-2 cells. These data suggest that IL-20 may be associated with tubular cell death, tubulointerstitial fibrosis, and renal inflammation in the progression of AKI. In addition, the expression of IL-20 showed a similar trend to serum creatinine and BUN levels, which suggests that IL-20 contributes to the severity of AKI [161]. Renal cell death, fibrosis, and inflammation are important in AKI-to-CKD transition. IL-20 may also contribute to the progression from AKI to CKD.

## 15. IL-20 in CKD

We found that the serum levels of IL-20 were increased in patients with stage 5 CKD compared with healthy controls. Additionally, the cellular sources of IL-20 are mesangial cells and macrophages in the kidney of patients with lupus nephritis. IL-20 expression is positively correlated with the severity of lupus nephritis, which suggests that IL-20 may participate in the development of lupus nephritis. In 5/6 nephrectomy-induced CKD rats, IL-20 is elevated in the kidney. The tubular epithelial cells, interstitial immune cells, and glomerular mesangial cells are the major cellular sources of IL-20 [166,168]. IL-20 promotes cell arrest at G0/G1-phase and induces cell apoptosis via caspase-3 and BAD in mouse tubular epithelial cells (TKPTS and M-1). Pro-fibrotic factor TGF-β is induced by IL-20 in interstitial fibroblast (NRK-49F) cells [166]. IL-20 increases the mRNA transcripts of MCP-1, CXCL10, CCL5, and IL-6, as well as the ROS and iNOS generation in mesangial cells through ERK 1/2 activation [168]. Previous studies reported that MCP-1, CXCL10, and CCL5 promote leukocyte recruitment. Oxidative stress plays an essential role in the pathogenesis of various kidney diseases [174,175]. ROS and iNOS inhibit cell growth and induce cell death. Based on the roles of IL-20 in CKD, we speculate that the infiltrating inflammatory cells, mesangial cells, or renal epithelial cells produce IL-20 and then trigger fibroblasts to produce more fibrogenic factors. IL-20 induces cell apoptosis in epithelial cells. TGF-β1 secreted by IL-20-stimulated fibroblasts further induces mesangial cells or fibroblasts to synthesize various matrix proteins and promote ECM synthesis, which eventually leads to ESRD. All of these events promote CKD progression.

## 16. IL-20 in DN

We previously reported that IL-20 was upregulated in the serum of patients with diabetes mellitus. The level of IL-20 is significantly increased in diabetic patients with kidney dysfunction. These data suggest that IL-20 participates in the pathogenesis of DN. In addition, the expression of IL-20 and IL-20R1 was increased in the kidneys of streptozotocin (STZ)-induced diabetic mice, which indicates that IL-20R1 signal might be critical for IL-20-mediated biological function in this model. Interestingly, we observed that IL-20 was not detected in conditionally immortalized murine podocytes; however, IL-20 was highly expressed in podocytes of STZ-induced diabetes mice, which raises the possibility that IL-20 might be upregulated under pathological conditions. We further found that hydrogen peroxide, high glucose, and TGF-β1 stimulate podocytes to secrete IL-20, which supports our hypothesis. We discovered that IL-20R1, IL-20R2, and IL-22R1 were expressed in conditionally immortalized murine podocytes, which indicates that podocyte is a target cell for IL-20. IL-20 enhances MCP-1, TGF-β1, MMP-9, and VEGF expression in podocytes through ERK, JNK, and p38 pathway. IL-20 promotes podocyte apoptosis through activating caspase-8 [167]. These data support the notion that IL-20 is involved in the progression of DN and may also contribute to the cascade of inflammation and diabetic glomerulopathy.

## 17. IL-20 Antibody Therapy in Kidney Disease

In 1975, Kohler and Milstein successfully manufactured B lymphocyte and myeloma cell fusion cells (hybridoma), which opened the application of monoclonal antibodies. Ten years later, the FDA approved the first monoclonal antibody drug, murine IgG2a CD3 (also called muromonab), to be used in organ transplantation. Currently, there are more than 100 FDA-approved antibodies for treatment. A lot of monoclonal antibodies are used in clinical trials of kidney diseases such as Adalimumab, Fresolimumab, and Rituximab [176]. Several animal experiments showed that blockade of IL-20 can effectively attenuate inflammation and ameliorate the severity of liver fibrosis [164]. Inhibition of IL-20 with specific antibody reduces renal tubular damage and decreases TGF-β1 and IL-1β production in the kidney of HgCl_2_-induced AKI rats [161]. In the DN model, neutralizing IL-20 decreases urine albumin/creatinine ratio and improves STZ-induced renal structure damages, including glomerular hypertrophy and mesangial cell expansion. Moreover, anti-IL-20 mAb ameliorates renal inflammation through reducing iNOS, TNF-α, and MCP-1 expression in STZ-induced DN mice. Our previous study showed that only IL-20R1 was increased in the kidneys of diabetic mice, which suggests that IL-20R1 may be necessary for IL-20-mediated DN. Blood urea nitrogen (BUN) and glomerular hypertrophy were improved in IL-20R1-deficienct mice [177]. Furthermore, anti-IL-20R1 mAb inhibits ROS production in mesangial cells and reduces the protein level of TGF-β1 in fibroblasts. Together, these data indicate that anti-IL-20 mAb might have a therapeutic potential to ameliorate kidney disease, including renal hypertrophy, inflammation, and fibrosis.

Fletikumab, a recombinant human anti–IL-20 mAb, has been tested in two clinical trials, psoriasis and RA [178]. Under the dose range from 0.05 to 3.0 mg/kg, Fletikumab was tolerable and non-toxic in patients with psoriasis. However, the clinical study was terminated because no apparent efficacy was observed. In the phase 2a trial, Fletikumab significantly reduced tender joint counts and swollen joint counts in seropositive RA patients. However, this clinical study ended at phase 2b because it failed to meet the primary endpoint [178]. We expect to see new clinical trials to validate the efficacy of using anti–IL-20 mAb for treating kidney diseases in the near future.

IL-20 shares receptors with IL-19 and IL-24; thus, they might have similar biological functions. The expression of IL-19 is upregulated in human renal proximal tubule cells (RPTEC/TERT1) treated with nephrotoxic agents. The mRNA level of IL-19 and its receptors IL-20R1/IL-20R2 is upregulated in the kidney, lung, and liver of mice that underwent IRI. IL-19 upregulates TGF-β1 and MCP-1 expression in renal cortical collecting duct cells (M-1). IL-19 also activates caspase-3 and caspase-9 to promote cell apoptosis through the p38 MAPK pathway in M-1 cells [179]. IL-20R1 deficiency ameliorates IRI-induced AKI. The role of IL-19 in DN is still unclear. The level of IL-19 is increased in uremia and DM patients. Moreover, the extent of IL-19 is positively correlated with the severity of urinary albumin excretion [180,181]. However, the mechanism of IL-19 in DN awaits further investigation. IL-24 is known as an anti-tumor cytokine for inhibiting cancer cell growth. The role of IL-24 in kidney disease is little known. Pap et al. found that renal IL-19 and IL-24 are increased in UUO newborn rats. TGF-β treatment inhibits IL-24 expression in HK-2 cells [182]. Besides, IL-24 reduces H_2_O_2_-induced-cell apoptosis by inhibiting caspase-3 activity and ROS generation in HUVECs. In hypertensive rats, IL-24 mRNA and protein levels are significantly decreased in kidney and increased after administration of anti-hypertensive drugs. According to these findings, IL-24 may be a protective cytokine in hypertension. Hypertension is an important risk factor for the development of CKD. However, the effect of IL-24 in kidney disease needs to be identified in the future [183].

## 18. Conclusions

**Figure 1:**
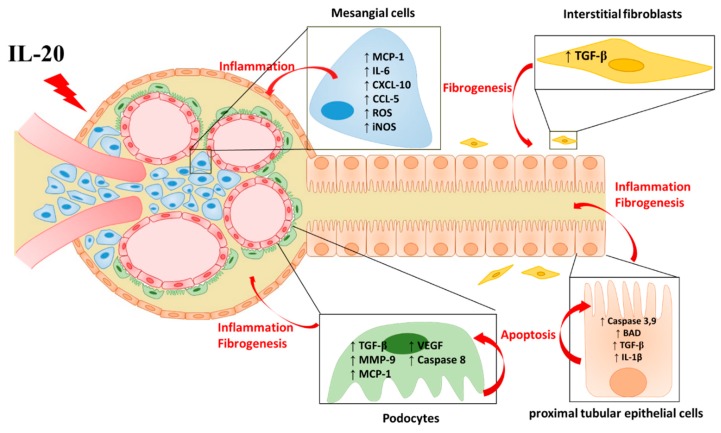
The effect of IL-20 in renal cells. IL-20 acts on interstitial fibroblasts, renal epithelial cells, mesangial cells, and podocytes and contributes to the progression of kidney disease, including inflammatory response, renal fibrosis, and cell apoptosis. ↑ = increased; ↓ = decrease.

In patients with kidney disease, renal inflammation and fibrosis commonly occur in tubulointerstitium with tubular atrophy, ECM accumulation, and loss of peritubular capillaries. Several factors participate in the development of the progression of renal diseases AKI, CKD, and DN. IL-20 acts on renal cells and contributes to inflammation, fibrosis, and apoptosis through its receptors IL-20R1, IL-20R2, and IL-22R1. We summarized the renal target cells of IL-20 and the possible regulating role of IL-20 in the pathogenesis of kidney diseases (Figure 1). In animal studies, we observed that blockade of IL-20 could improve renal function and structure. Therefore, we expected that therapeutic targeting of IL-20 might be beneficial to those patients in the future.

## Figures and Tables

**Table 1:** IL-20: biological effects and target cells.

Organ	Diseases	Target Cells	Role	Reference
Brain	Ischemic Stroke	Glia-like cells	↑ Inflammation ↑ Ischemic infarction	[162]
Mouth	Oral Cancer	Oral carcinoma cells	↑ Tumor progression ↑ Inflammation	[157]
Airway	Asthma	Lung epithelial cells	↑ Lung fibrosis	[160]
Arterial	Atherosclerosis	Endothelial cells	↑ Inflammation ↑ Angiogenesis ↑ Atherosclerosis	[158]
Liver	Hepatocellular Carcinoma (HCC)	Liver cancer cells	↑ Tumor progression	[163]
	Liver Injury	Hepatocytes	↑ Liver fibrosis ↑ Inflammation	[164]
Pancreas	Type 2 Diabetes	Pancreatic islets	↑ Inflammation	[165]
Kidney	Hgcl_2_-Induced AKI	Proximal tubular epithelial cells	↑ Inflammation ↑ Renal fibrosis ↑ Cell death	[161]
	5/6 nephrectomy-Induced CKD	Tubular epithelial cells Interstitial fibroblasts	↑ Renal fibrosis	[166]
	STZ-induced DN	Podocytes	↑ Inflammation ↑ Fibrosis ↓ Renal function	[167]
	Lupus Nephritis	Mesangial cells	↑ Inflammation	[168]
Skin	Psoriasis	Keratinocytes	↑ Cell proliferation	[169,170]
Bone	RA	Synovial fibroblasts Osteoclasts Osteoblasts Chondrocytes	↑ Inflammation	[155]
	Spondyloarthritis	Synovial fluid monocytes Synovial fibroblasts Osteoblasts	↑ Inflammation ↑ Osteoblastogenesis	[171]
	Osteoporosis	Osteoclasts Osteoblasts	↑ Osteoclastogenesis ↓ Osteoblastogenesis	[172]
	Osteoarthritis	Synovial fibroblasts Chondrocytes	↑ Inflammation ↓ Chondrogenesis ↑ Osteoblastogenesis	[156]
	Intervertebral Disc (IVD) Herniation	Disc Cells	↑ Inflammation	[173]

↑ = increased; ↓ = decrease.
